# Supplementary material for: Decision-making approaches used by UK and international health funding organisations for allocating research funds: A survey of current practice
Source: PLoS One. 2020 Nov 5;15(11):e0239757. doi: 10.1371/journal.pone.0239757 (PMC7644005; doi:10.1371/journal.pone.0239757)
Supplement: S1 File — Some questions would only be shown depending on prior answers. (DOCX) [file pone.0239757.s001.docx]

**Section 1. About you and your organisation**

We are a research team at the NIHR Evaluation, Trials and Studies Coordinating Centre (NETSCC).

We would like to invite you to take part in a global online survey to learn about the decision-making processes funders of health research use to allocate grant funds (for example, whether you use external peer reviewers and how many stages applications may go through). Your organisation has been identified as a health or health-related research funder and we would be interested in understanding more about your decision-making processes for research projects or programmes (not fellowships or infrastructure).

In Section 1, please tell us about you and your organisation.

Question 1.1

Name and email address (Note: by providing this information you are agreeing to us contacting you about this study and other peer review work undertaken by the NIHR. It will be stored separately to the survey data and will not be linked to your responses). You do not have to provide your contact details.

Name:

Email:

**Question 1.2**

What is the name of your organisation?

**Question 1.3**

What is your main role in the organisation? (e.g, programme director, research manager)

**Question 1.4**

What is the size of your organisation?

0-9 people

10-24 people

25-99 people

100-249 people

250+ people

I don't know

**Question 1.5**

Please answer the remaining questions based on one funding programme or theme (for research projects or programmes, not fellowships or infrastructure) that you are most familiar with (e.g. for NIHR Health Technology Assessment Researcher Led; or MRC Health Intervention Development Scheme; PHIND). Please be as detailed as possible.

If you have knowledge about more than one funding programme for your organisation then you can complete this questionnaire multiple times for each programme.

If your organisation doesn't distinguish funding by programme, please put the name of the fund.

Name of funding programme?

**Question 1.6**

What field of research does this programme fund? (e.g., clinical health research, health service evaluation, health technology)

**Question 1.7**

How many applications are typically received per round for the funding programme? (please use digits; e.g. 60)

**Question 1.8**

What is the success rate for the funding programme? (e.g. what percentage (%) of submitted applications are funded per round; please use digits (e.g. 20%))

**Question 1.9**

What is the average amount of funding per award in this funding programme? (please use digits and specify currency, e.g. £100000)

**Question 1.10**

What is the annual research budget of the funding programme? (please use digits and specify currency, e.g. £100000)

**Question 1.11**

Please indicate the core assessment criteria for informing decision-making for the programme that you identified, and rate for importance.

1 (not important)

2

3

4 (very important)

N/A

Remit and relevance

A need to generate evidence

Scientific rigour

Value for money

Low risk (e.g., study is feasible and likely to

produce outputs)

Quantitative methodological design

Qualitative methodological design

Innovation/originality of proposal

Timeliness (e.g. focus on current topics)

Experience of team/principal investigator

Award-holding institution

Engagement with relevant stakeholders

Potential research impact

Potential societal impact

**Question 1.12**

Are there any additional core assessment criteria (and how important are they) that are use to inform decision-making in this programme?

**Question 1.13**

Do core assessment criteria vary depending on the type of application?

Yes

No

**Question 1.13b**

If yes, please specify how the criteria vary:

**Section 2. Current processes for decision-making for grant fund allocation**

In the next section we would like to know about the current approaches your funding programme (identified in section 1) uses to facilitate decision-making for grant fund allocation.

**Question 2.1**

About the process: at how many stages does the researcher submit an application form (from when an application is first submitted to when a funding decision is made)? For example, if you have an expression of interest and then a full application there are two stages.

One stage applications

Two stage applications

Other

Not sure

**Question 2.1b**

If other, how many stages do you have?

**Question 2.2**

What approaches to decision-making are used within the stages/processes of the funding programme?

Yes

No

Not sure

Triage (only some applications go through to the next stage)

External peer review/Written review (experts in the field who are external to the organisation are sent applications to review)

Open peer review (reviewers comment publicly on applications)

Proportionate external/written peer review (where the number of reviewers are assigned according to the size of the grant or where reviewers are assigned certain sections of the application, based on expertise)

Face-to-face committee/board meeting

Flexible board/committee (different members attend each meeting based on expertise)

Virtual board/committee meetings (members 'meet' via telephone or an online platform)

Partial random allocation/lottery (applications to be funded are selected at random from a predefined pool)

Sandpits (groups of independent academics/clinicians/researchers are invited to collaborate on research projects)

**Question 2.3**

If you use triage, at how many stages can applications be given a non-fund decision? (For example, if you have a remit check, an expression of interest funding committee meeting, a full application funding committee meeting and a final sign off, this would be 4 stages).

One stage applications

Two stage applications

More/Other

Not sure

**Question 2.3b**

If other, how many triage stages do you have?

**Question 2.4**

If you use committee/board meetings, are decisions made using any of the following:

Mean scores

Consensus

Vote

Other

Not sure

**Question 2.4b**

If other, please specify:

**Question 2.5**

If you use different decision-making elements (e.g. an alternative to committee meetings or external/written peer review, for example), please specify:

**Question 2.6**

Is a scoring system used to aid decision-making?

Yes

No

**Question 2.6b**

If yes:

Is it a numeric or word score?

What is the scale?

Who provides the scores (e.g. external peer reviewers? committee members?)

How are the scores used? (e.g. at triage, by funding committee members, for feedback to applicants)

**Question 2.7**

What do you think are the main benefits of the current decision-making approaches for the funding programme?

**Question 2.8**

What do you think are the main drawbacks of the current decision-making approaches for the funding programme?

**Question 2.9**

Do you think that the current decision-making approaches used for the funding programme need to be improved?

Yes

No

Not sure

**Question 2.9b**

If yes, how do you think that they could be improved?

**Question 2.10**

For the funding programme specified, are you aware of any decision-making processes that are no longer used (e.g. external/written peer review, virtual committee meetings) and why these approaches are no longer used?

**Section 3. Alternative processes for decision-making for grant fund allocation**

In the last section we would like to know about approaches you and your organisation might try in the future to facilitate decision-making for funding allocation for your funding programme (identified in section 1).

**Question 3.1**

For the funding programme that you specified, please indicate whether the programme would like to use these approaches in the future.

Approaches to decision-making for fund allocation:

The organisation is planning to try it

I would like to try it

I don't like the idea

Triage (only some applications go through to the next stage)

External peer review/Written review (experts in the field who are external to the organisation are sent applications to review)

Open peer review (reviewers comment publicly on applications)

Proportionate external/written peer review (where the number of reviewers are assigned according to the size of the grant or where reviewers are assigned certain sections of the application, based on expertise)

Face-to-face committee/board meeting

Flexible board/committee (different members attend each meeting based on expertise)

Virtual board/committee meetings (members 'meet' via telephone or an online platform)

Partial random allocation/lottery (applications are selected at random from a predefined pool)

Sandpits (groups of independent academics/clinicians/researchers are invited to collaborate on research projects)

**Question 3.2**

If you have identified approaches that you would like to try, please specify why you would like to try these approaches:

**Question 3.3**

Have you or your organisation conducted any evaluations (e.g. audit, research studies, scoping) or are you aware of any evaluations or activities that support alternative approaches to external peer review and decision-making for funding allocation? Please specify.

**Question 3.4**

Are there any other comments you would like to add about decision-making for funding allocation in your organisation?

Thank you for taking part in this questionnaire.
