# Supplementary material for: Decision-making approaches used by UK and international health funding organisations for allocating research funds: A survey of current practice
Source: PLoS One. 2020 Nov 5;15(11):e0239757. doi: 10.1371/journal.pone.0239757 (PMC7644005; doi:10.1371/journal.pone.0239757)
Supplement: Attachment — Submitted filename: Response to Reviewers.docx [file pone.0239757.s004.docx]

Monday 7^th^ September 2020.

Dear Dr Visram

**Re: Revised Manuscript PONE-D-20-03358R1**

Thank you for your action letter on 19^th^ August 2020 regarding the manuscript: “Decision-making approaches used by UK and international health funding organisations for allocating research funds: A survey of current practice”. We were pleased to hear that R1 was happy that we had addressed the previous comments. We have responded to the review point made by R3 and uploaded a marked up and unmarked copy of our revised manuscript. Please find below our response to the review point. We hope that this revised manuscript now fully meets the PLOS ONE publication criteria*.*

Thank you and we look forward to hearing from you.

Kind regards,

Katie Meadmore

Senior Reseach Fellow

Wessex Institute, University of Southampton

**6. Review Comments to the Author**

**Reviewer #3: Thank you for the opportunity to review this paper. It is well written, methodologically sound and discusses an engaging issue well.

The comments to the author have been clearly addressed and result in a more rigorous paper.

Apologies for adding additional feedback, but one minor issue could be discussed in order to ensure full rigour. Your paper states that you took a mixed methods approach to data analysis, but then give only detail of the analysis for qualitative and quantitative strands as separate entities. There is no discussion of synthesis of the strands. If this was carried out, can it be detailed (e.g. was the analysis sequential or prioritised in any way)? If it was not, can this absence be justified in relation to your research question and design?**

Response: Thank you for taking the time to review our manuscript and for your comment. We agree that this detail was missing and have added in some text to clarify how we approached the mixed methods analysis in the data analysis section of the methods: *“A mixed methods approach to the analysis was taken in order to cross-validate findings and provide a fuller picture of the peer review landscape. Data were analysed separately but concurrently, and findings from each strand of data were triangulated to inform and explain patterns and interpretation. For example, qualitative data was used to expand and interpret quantitative findings and frequency data was used to confirm patterns in qualitative data.”*
